# An Unusual Complex Posterolateral Osteoligamentous Injury of the Knee in an Adolescent

**DOI:** 10.7759/cureus.7532

**Published:** 2020-04-04

**Authors:** Praveen Sodavarapu, Deepak Kumar, Aditya Guduru, Pratik M Rathod

**Affiliations:** 1 Orthopaedics, Post Graduate Institute of Medical Education and Research, Chandigarh, IND

**Keywords:** posterolateral corner, lateral collateral ligament, posterior cruciate ligament, iliotibial band, avulsion fracture

## Abstract

We present an unusual and complex case of a 16-year-old adolescent male who injured his right knee and sustained combined avulsion injuries of posterior cruciate ligament (PCL) at the tibial insertion site, iliotibial band at lateral tibial condyle, and lateral collateral ligament (LCL) at femoral insertion site akin to osteoligamentous posterolateral corner injury. Anatomical reduction of the femoral LCL avulsion fragment was performed and fixed with a two 4-mm partially threaded cancellous screw. Iliotibial band avulsion was buttressed using Ellis t-plate and fixed with two 4-mm partially threaded cancellous screws. PCL avulsion fracture was conservatively treated owing to minimal displacement. At one-year follow-up, the patient was pain free with a range of motion of 0 to 150 degrees of flexion and had a pain free knee with no instability. Posterolateral corner injury in the patient was very significant as it involved LCL avulsion and iliotibial band avulsion, both of which are part of the posterolateral structures of the knee and also involve the growth plate. Fixation of the avulsion of Gerdy's tubercle with the buttress plate helps to provide additional stability to counteract the deforming forces of the iliotibial band. LCL is also the major stabilizer against varus forces, and hence fixation is required for stability while preventing growth disturbance. PCL avulsion can be treated conservatively in those patients where the fragment is undisplaced or minimally displaced. A good outcome can be achieved in skeletally immature patients who have osteoligamentous posterolateral corner injuries with associated avulsion fractures by using appropriate anatomical reduction and surgical fixation.

## Introduction

Ligament injuries of the knee in adolescents are relatively uncommon and usually present as avulsion fractures [1]. Both lateral collateral ligament (LCL) avulsion and posterior cruciate ligament (PCL) avulsion injuries are infrequent in children and adolescents [2,3]. Iliotibial band avulsion from the lateral tibial condyle is an even rarer injury in this group of patients [4]. Such injuries can result in severe disability and can lead to degeneration of articular cartilage [5]. This article presents an unusual and complex case of a 16-year-old adolescent male who injured his right knee and sustained combined avulsion injuries of PCL at the tibial insertion site, iliotibial band at lateral tibial condyle, and LCL at femoral insertion site akin to osteoligamentous posterolateral corner (PLC) injury.

## Case presentation

**Figure 1:**
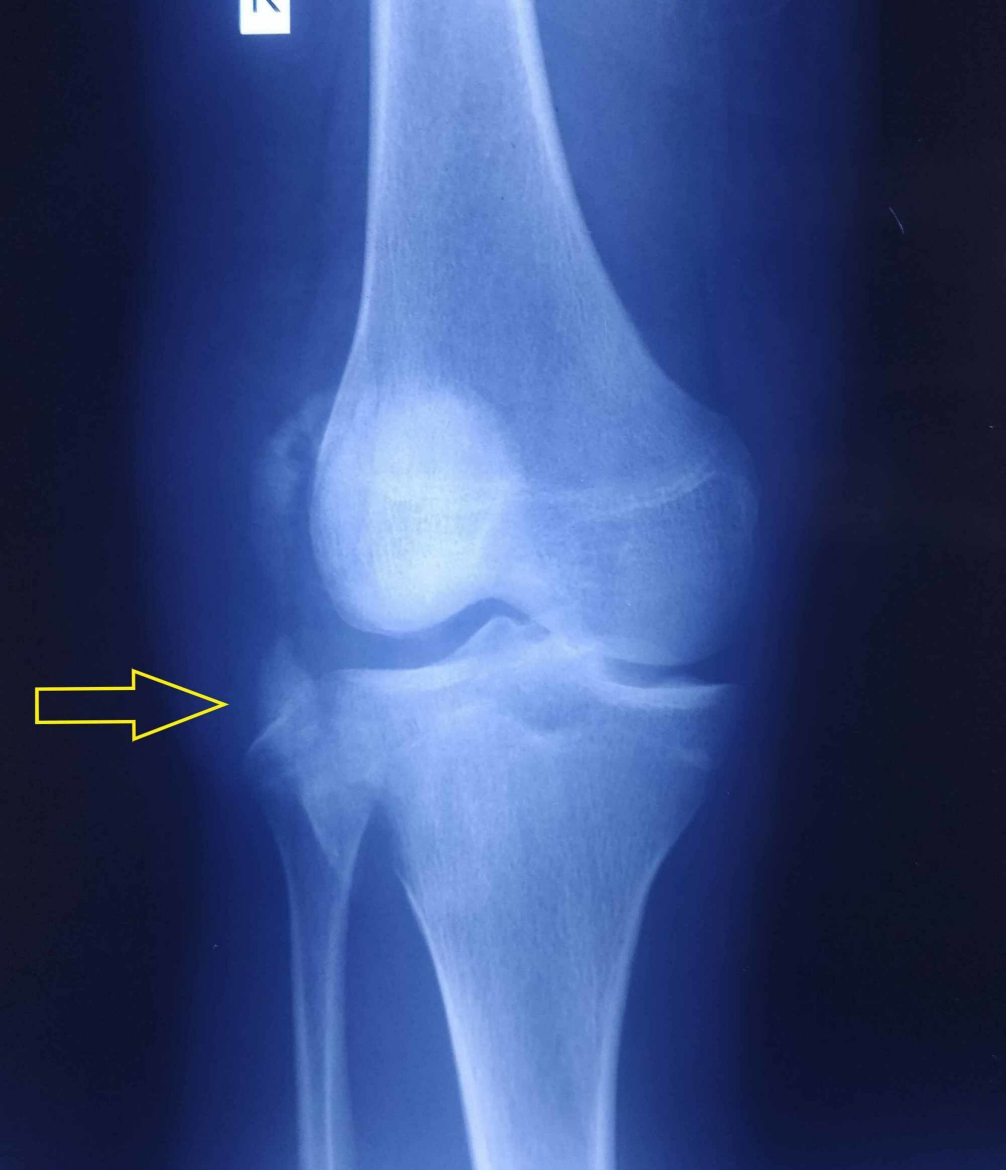
Preoperative anteroposterior radiograph of the knee showing a fractured fragment of the lateral tibial condyle (yellow arrow)

**Figure 2:**
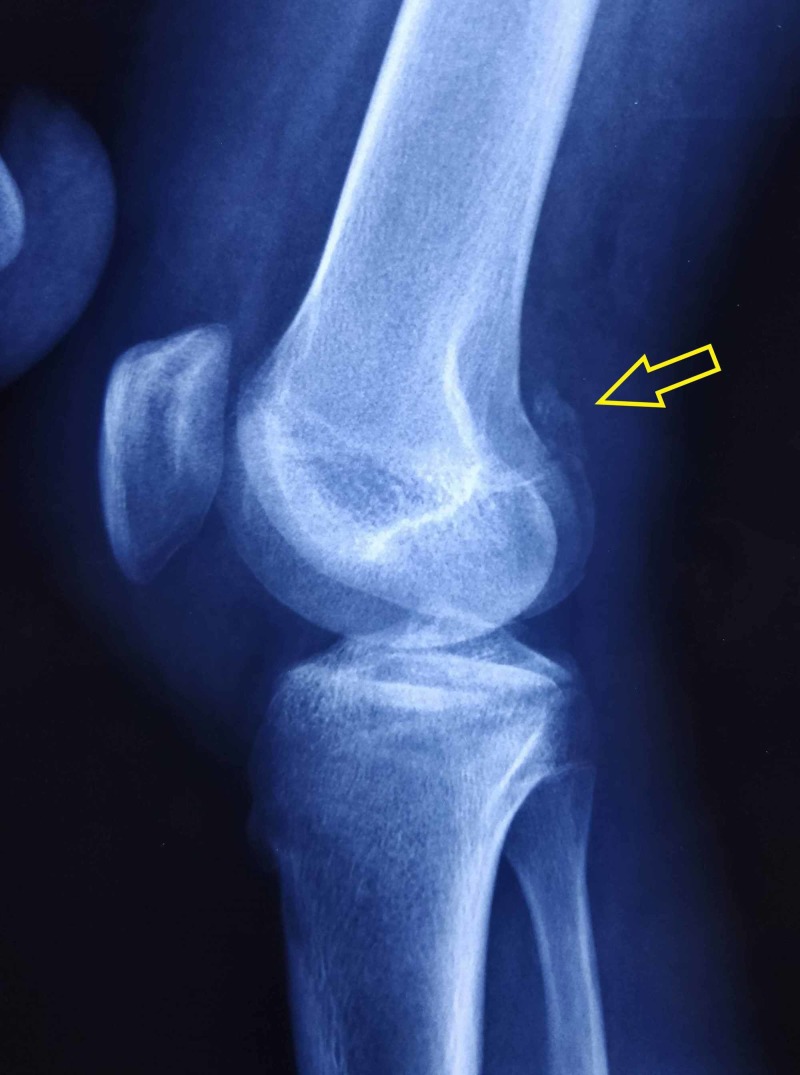
Preoperative lateral radiograph of the knee showing avulsed femoral fragment (yellow arrow)

A 16-year-old patient who met with a road traffic accident was brought to the trauma center with injury to his right knee and leg. On clinical examination, there was abnormal mobility and crepitus in his right leg and effusion of the right knee. Physical examination also revealed tenderness on the patella, the lateral aspect of the distal femur, and the lateral aspect of the proximal tibia. He had intact skin, motor, and sensory function, with palpable distal pulses and compressible soft compartments. Initial radiographs demonstrated a patella fracture and a tibial shaft fracture. Radiographs also revealed a large fragment along the lateral aspect of the knee with the fracture line extending from the tibial plateau into the metaphysis (Figures 1, 2).

**Figure 3:**
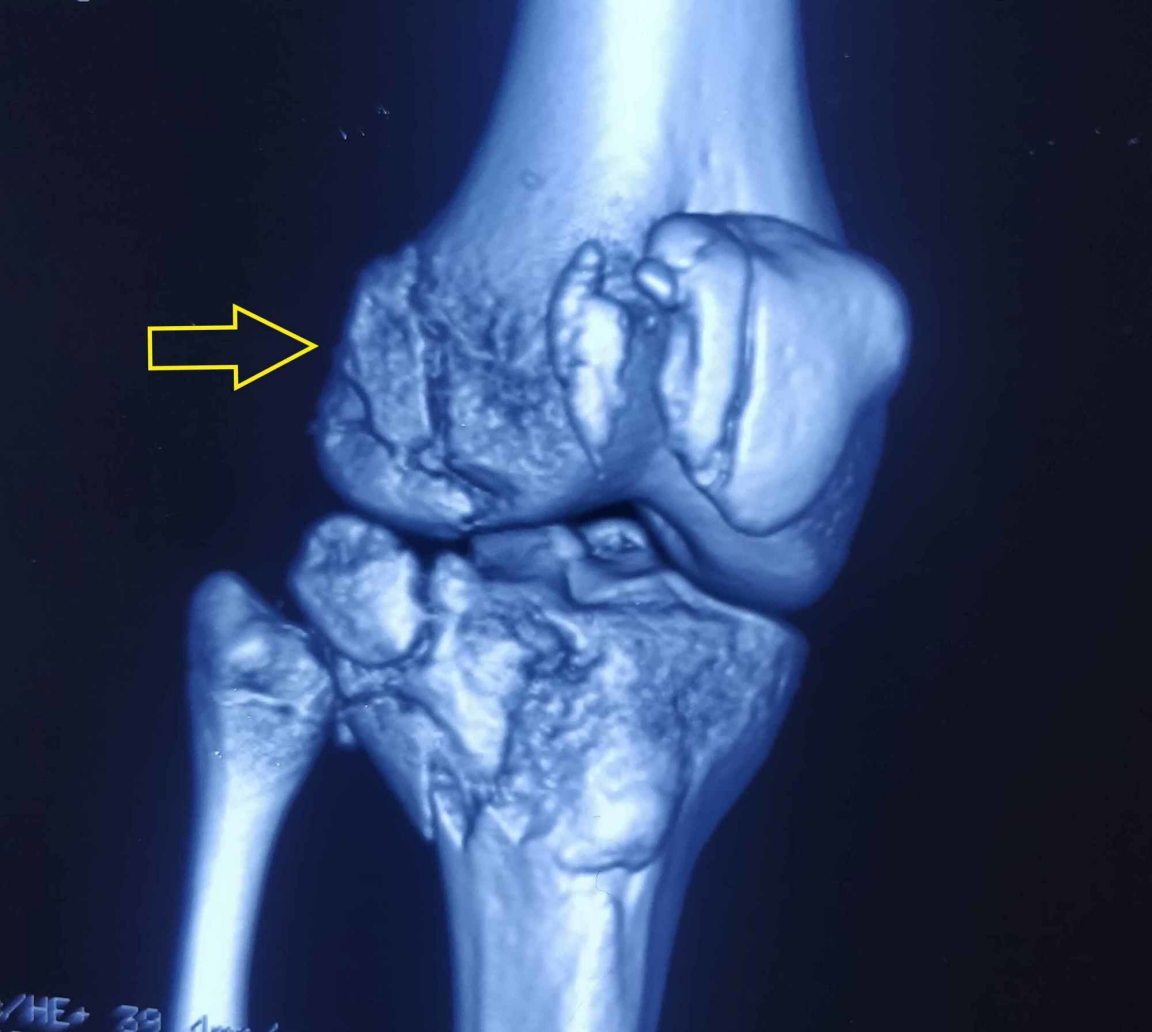
3D reconstruction image of CT scan showing femoral avulsion of posterolateral complex (yellow arrow)

**Figure 4:**
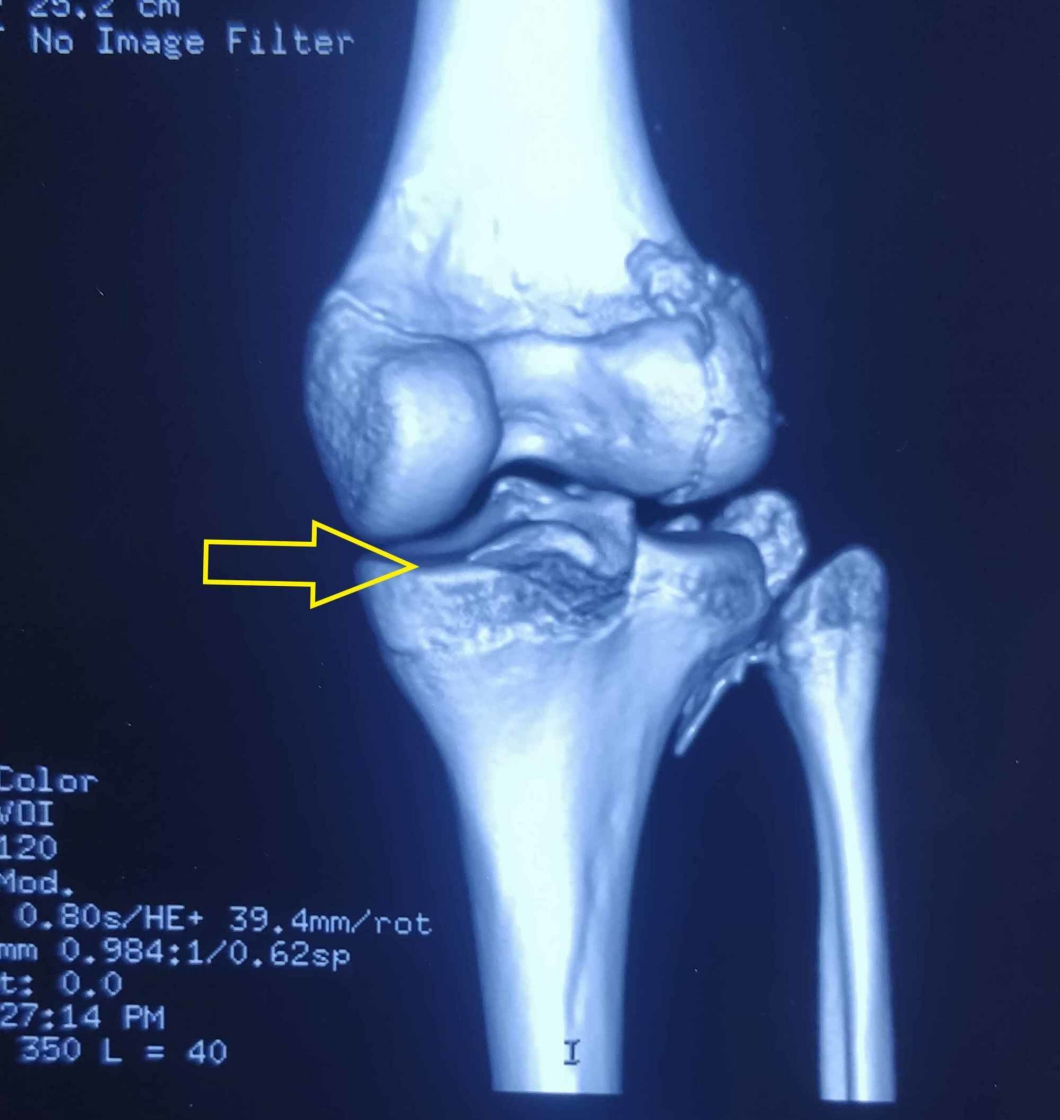
3D reconstruction image of CT scan showing PCL avulsion at the tibial insertion site (yellow arrow)

Computed tomography (CT) imaging was helpful in detecting the anterolateral location of the fragment, likely an avulsion of the iliotibial band from Gerdy's tubercle. CT also revealed a minimally displaced avulsion fracture of PCL and bony fragment along the lateral femoral condyle, which was possibly a femoral avulsion of the posterolateral complex (Figures 3, 4).

**Figure 5:**
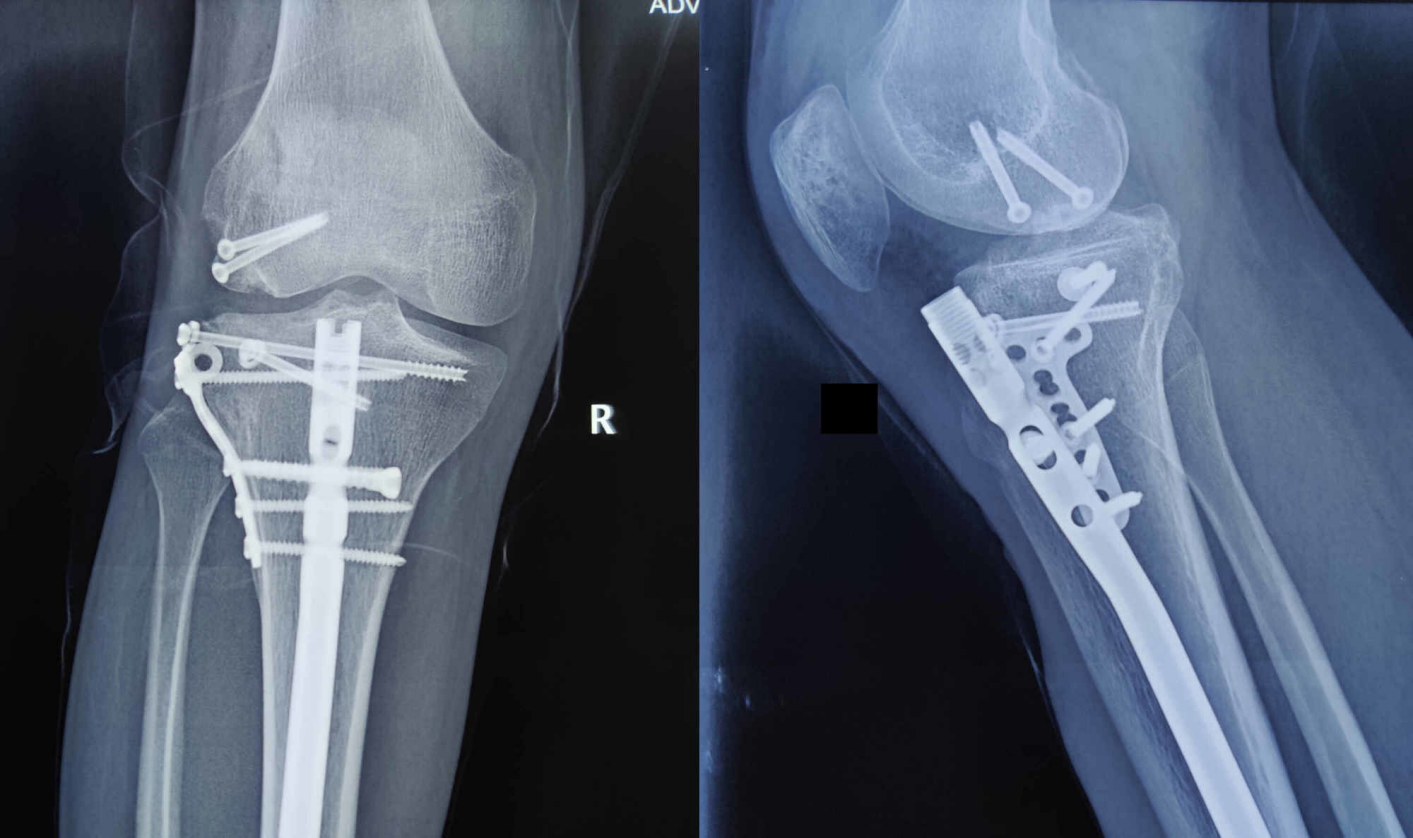
Anteroposterior and lateral radiographs of the knee at one-year follow-up

**Figure 6:**
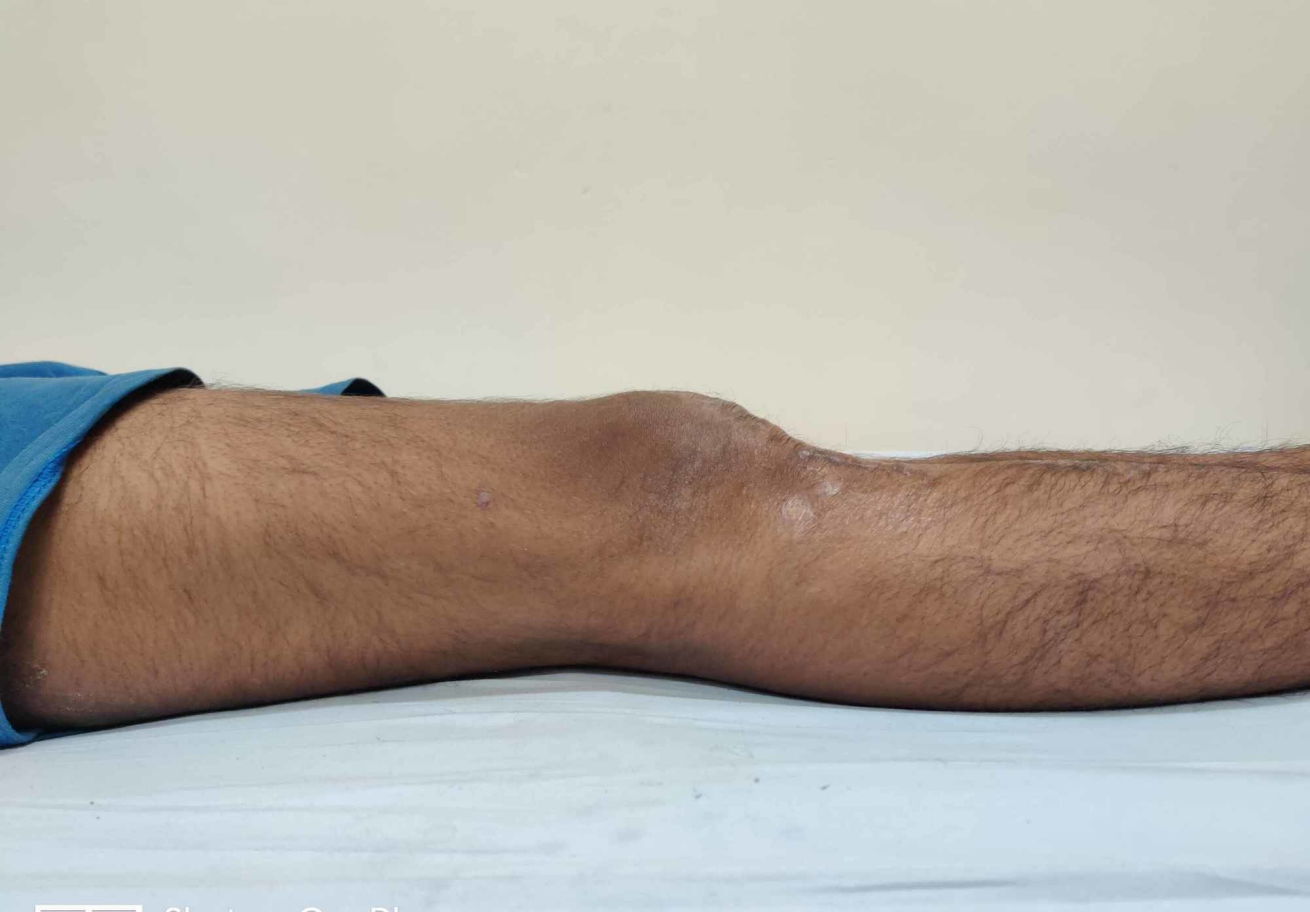
Clinical image showing a complete extension of the knee at one-year follow-up

**Figure 7:**
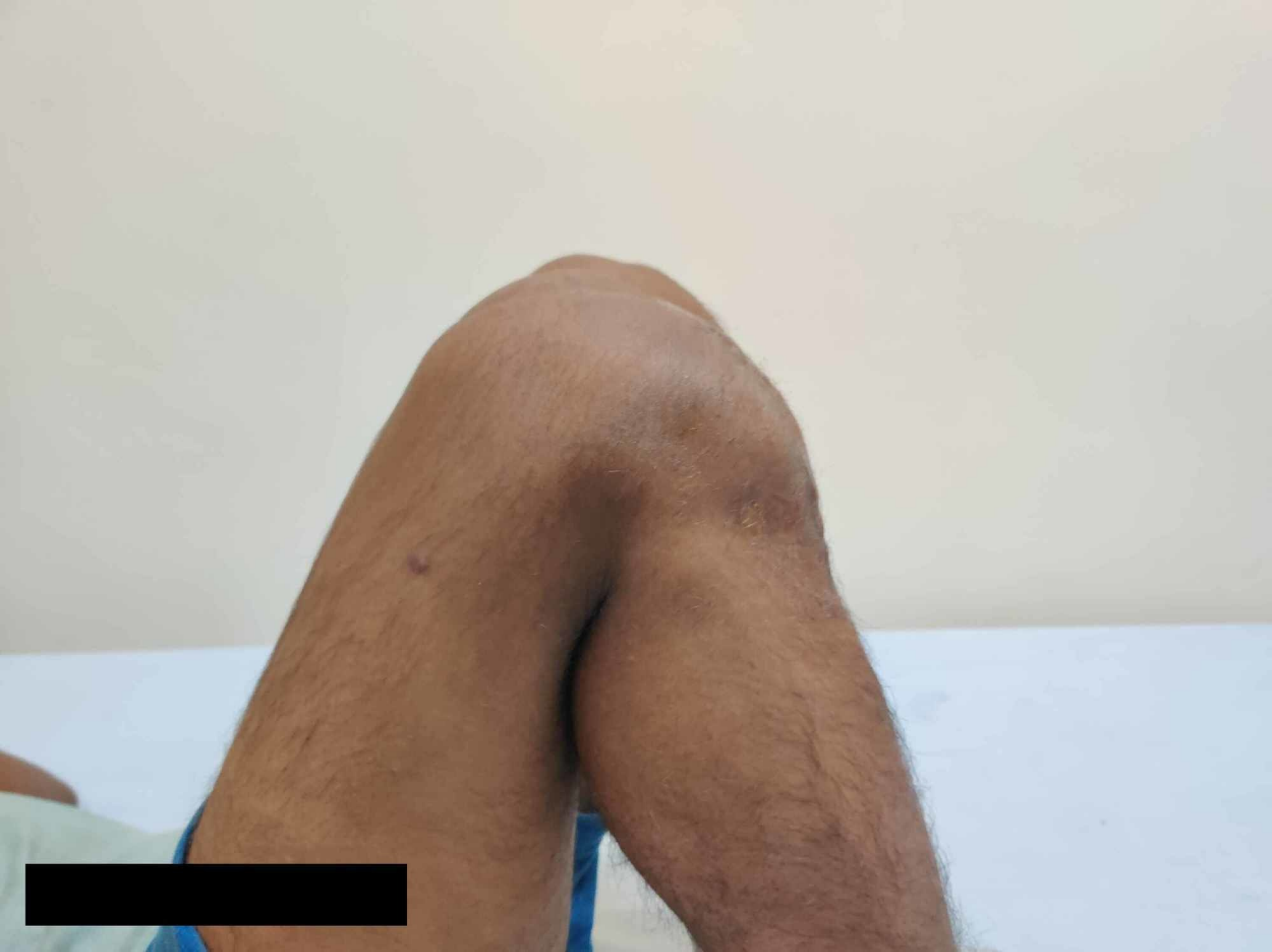
Clinical image showing flexion of 150 degrees of the knee at one-year follow-up

Based on the type of injury, a varus force has perceivably caused the avulsion fractures. Internal rotation of the tibia and hyperextension of the knee were speculated to have occurred based on the injury. Informed consent was taken from the patient for surgery. The patient was taken to surgery, and closed reduction with intramedullary nailing was performed for fracture of the tibial shaft. A longitudinal incision was used anteriorly over the knee to include the wound on the anterior aspect, and the fracture site was reached through the parapatellar approach. Dissection was performed up to the avulsed femoral fragment, which had the attachment of LCL. Attachment of popliteus tendon was intact over the femoral condyle. Anatomical reduction of the fragment was performed and fixed with two 4-mm partially threaded cancellous screws, and fluoroscopy helped to confirm the proper position of the screws to avoid violation of the growth plate. Then dissection was performed up to the fragment over lateral tibial condyle, which confirmed it as an avulsion of Gerdy's tubercle. The fragment was anatomically reduced and reattached to the anterolateral tibia using Ellis t-plate as buttress along with two 4-mm partially threaded cancellous screws. Patella was reduced and fixed using No. 2 non-absorbable braided suture. Because of minimal displacement, PCL avulsion fracture was conservatively treated. The wound was then irrigated, closed in layers, and the posterior splint was given with knee in 40 degrees of flexion. The postoperative period was uneventful, and the patient was discharged after one week. The splint was removed, and a knee brace was applied at the first postoperative visit. Partial weight-bearing and gentle range of motion (ROM) exercises were started after four weeks. He was allowed for a gradual increase in weight-bearing and ROM, and weaned out of the brace. The patient was serially followed up for up to one year, and after one year, the patient was pain-free with ROM of 0 to 150 degrees of flexion, had a stable knee with no instability noted with varus stress testing, external rotation stressing was comparable to opposite side, and the patient did not have any posterior sag on clinical examination. The patient was doing well, did not have any complaints, and has resumed his previous activity levels (Figures 5-7).

## Discussion

The avulsion fracture of the femoral attachment of the posterolateral structures is a rare injury in skeletally immature patients [6]. The main structures of PLC complex include the iliotibial tract, the LCL, the popliteus complex, the middle third of the lateral capsular ligament, the fabellofibular ligament, the arcuate ligament, the posterior horn of the lateral meniscus, the lateral coronary ligament, and the posterolateral part of the joint capsule [7-9]. The anatomy of these structures also may not be constant. LCL and popliteus tendon, as a part of the posterolateral complex, are the major stabilizers of the knee to varus and external rotation as determined from cadaveric studies [10-13]. Hence, such injury of LCL avulsion in our patient is considered as akin to osteoligamentous PLC injury. Ogden has described such avulsion fracture as a type 6 injury in his classification of physeal injuries and mentioned the possibility of growth disturbance when the peripheral part of the physis, which contains the node of Ranvier, is involved. Therefore, such injuries involving LCL attachment warrant fixation to avoid growth disturbances, in addition to providing stability [14]. LCL injuries are seen due to a combination of forces, including varus, extension, translation, and rotation, and may involve other structures of the PLC [15]. Acute injuries to the PLC in children and adolescents are unlikely but have been reported in a case series of six skeletally immature patients with avulsion fractures from the femoral attachment of the posterolateral structures of the knee. One patient who was treated non-operatively had valgus angulation, which required correction [6].

Injuries to the PCL are less common at all ages, especially when compared with those involving the anterior cruciate ligament (ACL) [16]. In skeletally immature knees, osteochondral avulsions are more likely than ligamentous tears due to strong ligaments when compared to physis [17]. Avulsion of PCL from the tibial insertion site is less frequent when compared to femoral avulsions [18]. In the absence of pertinent guidelines, the management of this injury in children and adolescents is influenced by the management of such injuries of ACL, which occur in adult patients. If the avulsed fragment of PCL attachment is not displaced or is minimally displaced, non-operative treatment can be suggested with good outcomes [19].

Avulsion fracture of iliotibial band from the lateral tibial condyle, specifically which involves the Gerdy's tubercle, is also an unlikely injury in children and adolescents. Very few cases have been reported with such injuries in this age group [4,20]. The iliotibial band exists as a part of the PLC, giving stability to the lateral aspect of the knee. The avulsion of Gerdy's tubercle involves both lateral tibial plateau and proximal tibial physis, and hence should be considered as a significant injury which requires anatomical reduction and fixation. Due to high pull forces acting over the avulsed fragment, screw fixation alone may not be sufficient, and hence buttress plating can provide additional stability to the fixation.

Sferopoulos et al. described seven patients with an avulsion fracture of the lateral tibial condyle in skeletally immature patients. Based on the anatomical location of the lesion, it was divided into two types. The Segond fracture was seen in five patients, involving the midportion of lateral tibial condyle just distal to the plateau. The avulsion of the iliotibial band at Gerdy's tubercle was seen in two other patients, which is bigger and anterior, and involves articular surface and proximal tibial physis. Conservative treatment was followed for these patients, and there were no complaints after nine years of follow-up. The author suggested surgical treatment if the avulsion fragment is large and involves the Gerdy's tubercle [5].

## Conclusions

In our patient, PLC injury was very significant as it involved LCL avulsion and iliotibial band avulsion, both of which are part of the posterolateral structures of the knee and also involve the growth plate. Fixation of the avulsion of Gerdy's tubercle with the buttress plate helps to provide additional stability to counteract the deforming forces of the iliotibial band. LCL is also the major stabilizer against varus forces, and hence fixation is required for stability while preventing growth disturbance. PCL avulsion can be treated conservatively in those patients where the fragment is undisplaced or minimally displaced. A good outcome can be achieved in skeletally immature patients who have osteoligamentous PLC injuries with associated avulsion fractures by using appropriate anatomical reduction and surgical fixation.

## References

[1] Kannus P, Jarvinen M (1988). Knee ligament injuries in adolescents. Eight year follow-up of conservative management. J Bone Joint Surg Br.

[2] Kannus P (1989). Nonoperative treatment of grade II and III sprains of the lateral ligament compartment of the knee. Am J Sports Med.

[3] Hurni Y, De Rosa V, Gonzalez JG, Mendoza-Sagaon M, Hamitaga F, Pellanda G (2017). Pediatric posterior cruciate ligament avulsion fracture of the tibial insertion: case report and review of the literature. Surg J (NY).

[4] Covey D (2001). Injuries of the posterolateral corner of the knee. J Bone Joint Surg Am.

[5] Sferopoulos NK, Rafailidis D, Traios S, Christoforides J (2006). Avulsion fractures of the lateral tibial condyle in children. Injury.

[6] Heideken JV, Mikkelsson C, Windhamre HB, Janarv PM (2011). Acute injuries to the posterolateral corner of the knee in children: a case series of 6 patients. Am J Sports Med.

[7] Seebacher JR, Inglis AE, Marshall JL, Warren RF (1982). The structure of the posterolateral aspect of the knee. J Bone Joint Surg Am.

[8] Sudasna S, Harnsiriwattanagit K (1990). The ligamentous structures of the posterolateral aspect of the knee. Bull Hosp Jt Dis Orthop Inst.

[9] Terry GC, LaPrade RF (1996). The posterolateral aspect of the knee: anatomy and surgical approach. Am J Sports Med.

[10] Nielsen S, Helmig P (1986). Posterior instability of the knee joint. An experimental study. Arch Orthop Trauma Surg.

[11] Nielsen S, Helmig P (1986). The static stabilizing function of the popliteal tendon in the knee. An experimental study. Arch Orthop Trauma Surg.

[12] Nielsen S, Ovesen J, Rasmussen O (1985). The posterior cruciate ligament and rotatory knee instability. An experimental study. Arch Orthop Trauma Surg.

[13] Nielsen S, Rasmussen O, Ovesen J, Andersen K (1984). Rotatory instability of cadaver knees after transection of collateral ligaments and capsule. Arch Orthop Trauma Surg.

[14] Ogden J (1990). Skeletal Injury in the Child. Saunders.

[15] Lee J, Papakonstantinou O, Brookenthal KR, Trudell D, Resnick DL (2003). Arcuate sign of posterolateral knee injuries: anatomic, radiographic, and MR imaging data related to patterns of injury. Skeletal Radiol.

[16] Allen CR, Kaplan LD, Fluhme DJ, Harner CD (2002). Posterior cruciate ligament injuries. Curr Opin Rheumatol.

[17] Parkkari J, Pasanen K, Mattila VM, Kannus P, Rimpela A (2008). The risk for a cruciate ligament injury of the knee in adolescents and young adults: a population-based cohort study of 46 500 people with a 9 year follow-up. Br J Sports Med.

[18] Pandya NK, Janik L, Chan G, Wells L (2008). Case reports: pediatric PCL insufficiency from tibial insertion osteochondral avulsions. Clin Orthop Relat Res.

[19] Coyle C, Jagernauth S, Ramachandran M (2014). Tibial eminence fractures in the paediatric population: a systematic review. J Child Orthop.

[20] Yoo JH, Hahn SH, Yang BK (2007). An en bloc avulsion fracture of tibial tuberosity and Gerdy’s tubercle in an adolescent basketball player: a case report. Knee Surg Sports Traumatol Arthrosc.

